# Innovative multi-material tool use in the pant-hoot display of a chimpanzee

**DOI:** 10.1038/s41598-022-24770-w

**Published:** 2022-11-29

**Authors:** Stuart K. Watson, Susan P. Lambeth, Steven J. Schapiro

**Affiliations:** 1 Department of Comparative Language Science, University of Zurich, Affolternstrasse 56, 8050 Zürich, Switzerland; 2 Center for the Interdisciplinary Study of Language Evolution, University of Zurich, Affolternstrasse 56, 8050 Zürich, Switzerland; 3 Department of Evolutionary Biology and Environmental Studies, University of Zurich, Winterthurerstrasse 190, 8057 Zurich, Switzerland; 4 Department of Comparative Medicine, The University of Texas MD Anderson Cancer Center, 650 Cool Water Drive, Bastrop, TX 78602 USA; 5 Department of Experimental Medicine, University of Copenhagen, Copenhagen, Denmark

**Keywords:** Psychology, Animal behaviour, Biological anthropology

## Abstract

‘Pant-hoot displays’ are a species-typical, multi-modal communicative behaviour in chimpanzees in which pant-hoot vocalisations are combined with varied behavioural displays. In both captivity and the wild, individuals commonly incorporate striking or throwing elements of their environment into these displays. In this case study, we present five videos of an unenculturated, captive, adult male chimpanzee combining a large rubber feeding tub with excelsior (wood wool) in a multi-step process, which was then integrated into the subject’s pant-hoot displays as a percussive tool or ‘instrument’. During the construction process, the subject demonstrated an understanding of the relevant properties of these materials, ‘repairing’ the tub to be a more functional drum when necessary. We supplement these videos with a survey of care staff from the study site for additional detail and context. Although care must be taken in generalising data from a single individual, the behaviour reported here hints at three intriguing features of chimpanzee communicative cognition: (1) it suggests a degree of voluntary control over vocal production, (2) it is a so-far unique example of compound tool innovation and use in communicative behaviour and (3) it may represent an example of forward planning in communicative behaviour. Each of these would represent hitherto undocumented dimensions of flexibility in chimpanzee communication, mapping fertile ground for future research.

## Introduction

Understanding the cognition underlying animal vocal behaviour not only provides crucial insights into the ecology of a species, but can also provide powerful comparative data for understanding the evolution of human cognition^1^. For instance, data on primate vocalisations has shed light on features of cognition relevant to language evolution including intentionality^2,3^, combinatoriality^4–10^ and semantics^11–13^. However, the study of vocal behaviour can also have relevance to other domains such as social cognition^14–17^, memory^18^, and cultural transmission^19–24^. Indeed, even observations of a single individual from a species can generate important insights into the cognitive constraints in which a species exists^25–30^, even if the specific conditions and behaviours in question are not generalisable^31,32^. Here we present a set of observations of an interesting behavioural innovation in the ‘pant-hoot displays’ of a captive male chimpanzee (*Pan troglodytes*) which provides fresh insights into the complexity of cognition underlying communicative behaviour in chimpanzees.

Pant-hoots are a long-distance vocalisation produced by chimpanzees, typified by an introduction (a series of ‘hoo’-like vocalisations), a build-up (similar to an increasingly rapid panted-grunt), a climax (one or more scream-like vocalisations) and sometimes a ‘let-down’ phase (similar in structure to a build-up but with decreasing pace)^33^. This behaviour often coincides with other visual cues such as piloerection and a facial expression known as ‘pout face’^34^. This call type is deployed in a number of contexts for various functions including group coordination^35^, group movement^35^, strengthening social bonds^36^, feeding^5,35–37^ and dominance displays^38^. During the production of these calls, wild individuals regularly combine their pant-hoot with behaviours that interact with the caller’s environment, such as drumming on the buttresses of trees^39^, dragging branches^40^ and throwing rocks^41^. Although the function of interacting with these materials is little understood, a reasonable assumption is that they augment the vocal component of the signal—perhaps providing contextual information to receivers^42^, or simply increasing the saliency of the display^38,43,44^. Recent work even indicates that the timing and rhythmic properties of buttress drumming may encode information about the identity of the signaller^45^. Incorporation of such behaviours into pant-hoot displays is also common in captive chimpanzees, where individuals may have a diverse range of natural and artificial objects and materials in their enclosures to take advantage of^46^.

Here, we present five recorded observations of one captive, group-living, adult male chimpanzee who incorporated multiple objects into a multi-step preparatory phase of their pant-hoot displays (available to watch at: https://osf.io/w68fr/). Specifically, our study subject (‘EH’) combines a large plastic tub (65 cm wide x 25 cm deep) with ‘excelsior’ (i.e. wood wool) into a stack (hereafter a ‘tub-stack’), which EH then strikes before the climax phase of their display. We supplement these observations with further anecdotal data from experts working at the study site (care staff, trainers, and veterinarians) regarding their observation of this and other, similar behaviours at the study site. The finer details and timing of various aspects of the tub-stack behaviour may reveal several interesting features of chimpanzee cognition, including voluntary control over vocal production, innovative material use and forward planning.

## Methods

### Study individual

Our focal subject was individual ‘EH’, an adult male chimpanzee from the sub-species *Pan troglodytes verus* (aged 24 years) housed in a group of 8 other chimpanzees (3 male, aged 24–36. 5 female, aged 33–50) housed at the National Center for Chimpanzee Care located at the Michale E. Keeling Center for Comparative Medicine and Research of The University of Texas MD Anderson Cancer Center (UTMDACC) in Bastrop, Texas. The group had access to indoor spaces (two “dens” of 14 m2 each) and an outdoor (400 m2) enclosure with a range of enrichment devices and activities, and a variety of climbing and swinging structures to promote species-typical behaviours, which they were able to move freely between throughout the study period. Biscuits and fresh produce were provided to the chimpanzees two and three times per day respectively, water was available ad libitum and fresh nesting material (excelsior) was provided daily. According to keeper reports, EH was the dominant male at the time of our recordings. EH was born in captivity at another facility and raised by their biological mother, who was not present in the group at the time of the study. EH had participated in numerous behavioural experiments prior to these observations, including two run by SKW^47,48^, the researcher who recorded the videos described here. These experiments were unrelated to vocal behaviour or drumming, and did not involve any of the materials used in the tub-stack, nor was this behaviour observed during their course.

### Video recording and coding

Video footage was recorded using a Sony HDR-CX240E handheld digital video recorder at 25 fps. Videos were viewed and coded using BORIS software (version 7.13.9)^49^. These observations were recorded during the habituation phase of the experiment described in Watson et al.^48^. In brief, the group were habituated to predictable sequences of different acoustic tones (audible in the videos attached as supplemental material here), of which their ability to recognise and process would later be tested in a ‘violation of expectation’ paradigm. This involved setting up a speaker in the group’s indoor enclosure for 30 min, twice per day for 5 days and playing the stimuli. SKW was the only human present during these sessions, but the chimpanzee groups were free to come and go. EH was typically alone during these observations (though other individuals are present in Video 4), but the rest of the group were a short distance away through a doorway, well within hearing distance. The tub-stack behaviour was not observed in any of the other 24 subjects from Watson et al.^48^, and the researchers did not observe any influence of the habituation sounds on vocal or any other behaviours. All videos described here, with their corresponding BORIS project files, coding schema and outputs can be found at the following repository: https://osf.io/w68fr/.

### Ethics

Ethical approval for this study was granted by the Institutional Animal Care and Use Committee of the UTMDACC, adhering to all the legal requirements, guidelines and regulations of U.S. law and the American Society of Primatologists’ principles for the ethical treatment of nonhuman primates. Regarding our staff survey, participation was voluntary and informed consent was obtained.

## Results

### Tub-stack observations

**Figure 1:**
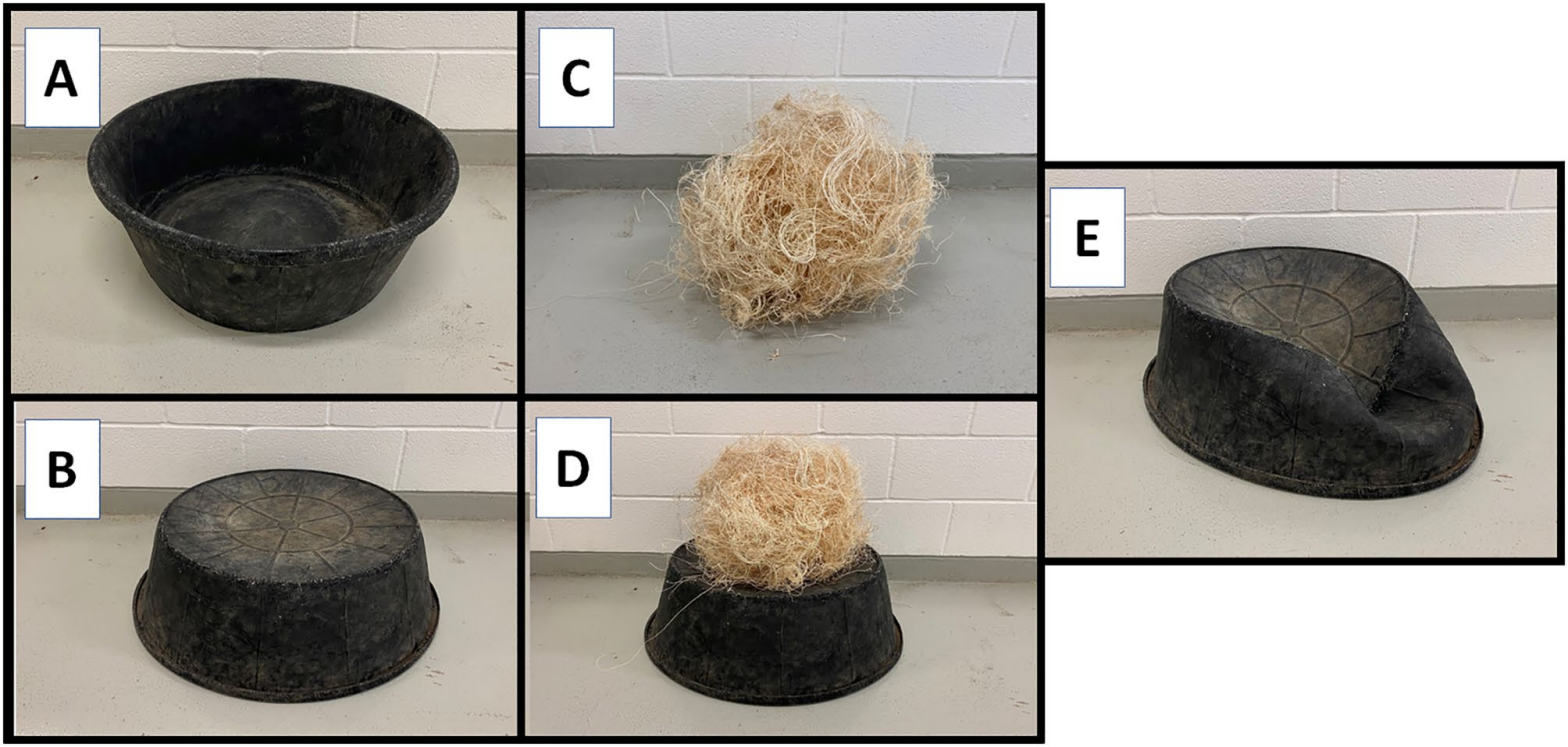
(**A**) Face-up tub. (**B**) Face-down tub. (**C**) Excelsior. (**D**) Excelsior placed on tub (‘tub-stack’). (**E**) Broken tub.

The tub-stacking behaviour was directly recorded on video on five occasions (Videos 1–5, Table 1) and observed on at least two other occasions (audio recorded—see videos 6 and 7, Table 1) over 5 days. SKW observed the behaviour on one other occasion 2 weeks after these recordings, while no experiment was ongoing, but this was not recorded and notes were not taken. While there is variation between videos, the broad behaviour of interest is the combination of a rubber ‘tub’ (typically used as a feeding tub, Fig. 1A,B) and ‘excelsior’ (wood wool provided as a bedding substrate, Fig. 1C) into a compound object we refer to as a ‘tub-stack’ (Fig. 1D). Tub-stack construction was either concurrent with or followed by (either immediately or after a short pause) a pant-hoot display, during which the subject produced a pant-hoot and struck the tub-stack at least once. In every observation, the tub was struck directly before the climax (scream) element of the pant-hoot. After striking the tub-stack, the tub itself was typically left in a dented state which rendered it less viable for use as a drum (see Video 5, Fig. 1E). It was therefore often necessary for EH to ‘repair’ the tub before creating the tub-stack: This involved flipping the tub, pushing out the bottom and then flipping it again (as shown in Video 2, see Table 1). We note that EH also frequently performed pant-hoot displays that did *not* include the tub or excelsior in any capacity (See Videos 8–10 for examples). Moreover, even within the few recorded observations of the tub-stack, it appears there may be interesting variation in behaviour (Table 1). The tub-stack behaviour, therefore, seems to be flexible in its deployment (i.e. whether EH chooses to use it or not) and structure (i.e. whether it includes a repair step, and the timing of vocal onset), rather than being a highly ritualised part of EH’s pant-hoot display behaviour. However, with a limited dataset it is not possible to determine whether this variation is statistically robust.

**Table 1:** Annotated events with timestamps for each recording of the tub-stack pant-hoot behaviour, which can be downloaded from https://osf.io/w68fr/.

Video 1		Video 2		Video 3		Video 4	
Time (s)	Behaviour	Time (s)	Behaviour	Time (s)	Behaviour	Time (s)	Behaviour
5.7	Pick up tub	11.27	Touch tub	1.7	Pick up tub	4.0	EH leaves view
7.0	Put down tub	12.2	Pout-face begins	1.7	Touch tub	8.0	Touch tub (out of view)
7.5	Touch tub	12.4	Pick up tub	8.7	Put down tub	9.0	Repair tub (out of view)
16.4	Pout-face begins	13.2	Repair tub	8.7	Pushes tub towards corner	11.2	Touch tub (out of view)
16.7	Excelsior placement	16.2	Put down tub	9.0	EH leaves frame	12.0	EH enters view
17.4	Stop touching tub	16.2	Stop touching tub	11.0	Excelsior placement (out of view)	12.7	Put down tub
18.4	Vocalising begins	17.4	Excelsior placement	14.0	Non-focal enters room	12.7	Stop touching tub
19.7	Excelsior placement	19.9	Excelsior placement	15.0	EH enters view	14.4	Excelsior handling
23.1	Pout-face ends	20.7	Touch tub	16.6	Touch tub	15.5	Vocalising begins
22.5	Strike tub	21.2	Stop touching tub	20.1	Stop touching tub	17.0	Excelsior placement
23.0	Jump and strike wall	25	Excelsior handling	31.0	EH leaves view	17.5	Strike tub
24.4	Vocal climax	37.7	Vocalising begins	33.0	Possible EH vocalising	17.7	Vocal climax
25.1	Vocalising ends	43.2	Strike tub	38.0	Possible EH vocalising	19.8	Strike wall
25.4	Strike tub	44.2	Vocal climax	41.9	Non-focal enters room	20.8	Vocalising ends
26.1	Strike tub	46.2	Strike wall	71.3	Vocalising begins		
26.9	Strike tub	46.2	Pout-face ends	76.0	EH enters view		
27.9	Strike tub	47.1	Vocalising ends	76.1	Strike tub		
28.4	Vocalising begins			77.3	Vocal climax		
28.9	Vocalising ends			78.6	Vocalising ends		
28.9	Strike tub						

Video 5		Video 6		Video 7		General notes	
Time (s)	Behaviour	Time (s)	Behaviour	Time (s)	Behaviour		
3.0	EH leaves view	02:00	Excelsior placement	07:00	Excelsior placement	Video 2: Clear footage of "repair" behaviour in this video	
6.0	Excelsior handling audible	08:00	Vocalising begins	16:00	Vocalising begins		
12.0	EH enters view	12:00	Strikes tub	21:00	Strikes tub	Video 3: Uncertain vocaliser at 33 s and 38 s—may be beginning of pant-hoot	
22.9	Vocalising begins	13:00	Vocal climax	22:00	Vocal climax		
28.7	Strike tub (gently)						
29.2	Strike tub (gently)					Video 4: Repair behaviour occurs out of frame at approx 8 s	
29.4	Strike tub (gently)						
30.2	Climax						
30.2	Vocalising ends					Video 5: Tub is dented and EH does not "repair". May explain use of more gentle strikes in this video. Excelsior displaced at 6.0 is visible to left of tub	
						Video 6 and 7: EH is out of frame but audible	

Two additional videos (Videos 6 and 7) are included in the repository in which the tub-stacking behaviour is taking place out of frame—no detailed notes exist for these incidents, but we include them for interested readers. Videos 8, 9 and 10 (not annotated) in the repository depict a ‘typical’ pant-hoot display which does not include the tub-stacking behaviour.

In Table 1 below, we annotate the events of each video. We strongly recommend that readers watch the videos for themselves (https://osf.io/w68fr/) and simply use our annotations as a guide towards moments of interest.

### Survey of expert staff at study site

We consulted three expert members of staff from UTMDACC, each with over a decade of experience at the site who had known and worked closely with EH for a collective 45 years. They, therefore, have a deep familiarity with the subject and their behaviour, as well as the other chimpanzees living at the study site. The full answers (minus any identifying information) can be found in our data repository (https://osf.io/w68fr/), which we summarise below.

1. **Have you (or anyone you have observed) ever knowingly rewarded the tub-stacking behaviour?**

None of the staff surveyed had observed the tub-stacking behaviour, before or after the date of recordings. Therefore, all agreed that they had never knowingly rewarded the behaviour, nor observed another human do so. This may indicate that the tub-stack behaviour was a recent innovation at the time of recording, and does not seem to have been copied by any other individuals (perhaps because of EH’s position as dominant male). EH was moved to another site in September 2019, 10 months after our tub-stack observations were recorded.

(2) **Can you think of any reason a keeper or trainer would have created something the 'tub-stack', or anything like it, in front of the chimpanzees?**

The staff surveyed unanimously agreed that there was no reason EH or any other chimpanzee would observe a human creating a tub-stack or similar object. This was almost impossible, because feeding tubs were always either inside the enclosure with the chimpanzees (which humans could not enter) or were out of sight while staff cleaned and restocked enclosures, during which the chimpanzees were locked in a separate area. All also agreed there was no reason staff would create a tub-stack like object for EH to find independently. It is therefore extremely unlikely that EH learned the behaviour by learning it from a human, or by recreating the product of a human.

(3) **Have you ever observed the tub-stacking behaviour in another individual at the study site?**

All staff agreed that the tub-stacking behaviour had not been observed in any other chimpanzees at the site. This indicates that it was an idiosyncratic behaviour innovated by EH, rather than learned through observation of others.

(4) **Have you observed any similar behaviours, perhaps using other objects or materials, in the population?**

Although no other individuals at the site were reported to use the tub-stacking behaviour or combine materials in a similar fashion, there were reports of potentially related behavioural forms. Some individuals (including EH) were frequently observed to incorporate large (~ 200 L) empty barrels into their pant-hoot displays (pushing them around and striking or drumming on them), which were often brought to a particular spot in the enclosure before the pant-hoot was initiated. Unlike the tub-stacking behaviour, this did not involve any combination or ‘repair’ of materials. One other individual at the site was reported to strategically choose the noisier of two objects (balls which were either empty or contained noisy rattling objects) to throw at the wall during their displays. Taken together, these behaviours would indicate that sensitivity to the acoustic properties of objects and their corresponding utility to a pant-hoot display is relatively widespread in captive chimpanzees. This is consistent with data from the wild, in which chimpanzees were found to target their drumming behaviour towards tree buttresses with wide, thin buttresses^50^, and likewise target their stone-throwing behaviour towards trees that produce less damped impact sounds^44^.

## Discussion

Here we have presented a set of observations of one captive, group-living, male chimpanzee who combined multiple materials (a feeding tub and excelsior, Fig. 1) into a single composite object (a ‘tub-stack’) which was then used percussively in EH’s pant-hoot displays. While this behaviour was restricted to a single individual, we believe it can nevertheless shed light on the cognition underlying pant-hoot display behaviour, adding to the growing literature demonstrating flexible control over communicative behaviour in chimpanzees^51^. More specifically, we argue that the behaviours reported here may illuminate three dimensions of chimpanzee cognition: voluntary control over vocal production, innovative material use in communicative behaviour, and forward planning.

Before exploring the interesting properties of our data, it is important to highlight the limitations inherent to a case study such as this. Foremost is that the limited number of observations makes it susceptible to various sampling biases, meaning that elements which exist in these videos may not be representative of the majority of out-of-sample incidents and likewise, common elements in unobserved examples may not be present here. We therefore hope that readers will join us in interpreting this data with both generosity and caution, using it as an intriguing starting point for generating hypotheses and further work on related topics, while also taking care not to overgeneralise.

### Voluntary control over vocal production

Production of vocal behaviour in primates has been argued by some researchers to be strictly governed by internal arousal states, in large part due to the finding that apes are lacking key neural circuitry which is known to be important for the voluntary control of vocal production in humans^52–54^. However, there is also a growing body of evidence demonstrating hitherto unsuspected flexibility in the vocal behaviour of apes^51,55,56^, to which EH’s tub-stacking behaviour may contribute a further data point. Specifically, we suggest that because in three out of four observations of a completed tub-stack (Videos 1–4) EH initiated vocal production after the condition of a completed tub-stack is satisfied, this indicates that they possessed some degree of control over the moment of deployment of a pant-hoot display.

It seems unlikely to us that EH has total control over the onset of pant-hoot displays, as it is visible in Videos 1, 2 and 4 that the motivational state underlying pant-hoot display behaviour has its onset before EH has finished building the tub-stack (indicated by the ‘pout’ facial expression that often precedes pant-hoots). However, in only one case (Video 4) did EH begin to vocalise before the tub-stack was complete (Video 4). This likely indicates that EH is able somehow to suppress the onset of the pant-hoot vocalisation itself. We also highlight Video 3, where the pant-hoot does not occur until almost a full minute after the tub-stack is created. In this example, we find it plausible that the onset of the display is under top-down control. However, it is also plausible that EH’s motivational state was simply disrupted by the entrance of their group mates to the chamber (timestamp—00:41) and was later resumed. In this case, this may reflect recent findings that chimpanzee signallers are sensitive to the presence and absence of specific audience members, altering the production and structure of their pant-hoot displays accordingly^38^. In Table 2 below, we propose and evaluate three alternative, speculative accounts which may explain the observed pattern of behaviours in terms of voluntary control. While account 3 has the least contradictory evidence, none of these accounts seem able to readily explain the behaviour in all videos, and it may therefore be wrong to consider them mutually exclusive. In other words, some episodes of pant-hoot display behaviour may be deployed voluntarily while others may result from non-voluntary arousal states (as humans may laugh reflexively or voluntarily, depending on the specific context).

**Table 2:** Three speculative accounts for the incorporation of tub-stacks into pant-hoot displays. The accounts are non-exhaustive, and not mutually exclusive.

Account	Description
**Subject possesses top-down control over vocal production**	EH is able to produce pant-hoot displays at-will. He is therefore able to deploy the pant-hoot display behaviour at any moment
In favour:	Long pause between completion of tub-stack and pant-hoot behaviour in Video 3
Against:	Overlap of pant-hoot behaviour (e.g. 'pout-face') and tub-stack creation in Videos 1, 2 and 4 suggests the onset of former is not under strict control
**Subject can anticipate future motivational state:**	Pant-hoot display production is tied to a particular corresponding motivational state. EH anticipates this impending motivational state and creates tub-stack in preparation
In favour:	Beginning of tub-stacking behaviour appears to precede pant-hoot related behaviours (e.g. vocalising, pout-face) in all videos
Against:	The overlap between pant-hoot display state and tub-stack creation in videos 1, 2 and 4 indicates not entirely anticipating the pant-hoot display state. Any anticipation may therefore be very short-term
**Subject can suppress current motivational state**	Pant-hoot display production is tied to a corresponding particular motivational state, but it can be suppressed for long enough to create tub-stack
In favour:	Broad overlap of 'pout-face' with tub-stacking in Videos 1 and 2, but calling never begins until tub-stack is complete
Against:	Break between tub-stack creation and pant-hoot display in Video 3 indicates lack of overlap in motivational states. But may be explained by EH being distracted by new individuals entering room

### Innovative material use in communicative behaviour

We argue that the tub-stack behaviour represents a case of innovation involving the combination of materials applied to a communicative behaviour. While innovations are not particularly uncommon in chimpanzees, especially captive individuals^57,58^, the tub-stacking behaviour represents an interesting data point for two reasons.

Firstly, tub-stacking represents a rare case of spontaneous multi-material tool construction (combination of more than one material to create a useful object^59^) in a non-human ape. Captive chimpanzees and orangutans have also both been found to use sticks to push down and then ‘fish out’ sponge materials to access juice rewards^60,61^, but these behaviours were heavily scaffolded under experimental conditions. The authors also frame these behaviours as ‘sequential’ tool-use, where separate materials are used one after another, rather than compound tools, where multiple materials are combined into a single object. Multi-material tools are also used by chimpanzees in the wild—for example, combining leaves and moss for ‘leaf-sponging’^62^, or a rock and piece of wood to act as hammer and anvil for nut-cracking^63^. However, to our knowledge, the tub-stack represents the first example of a multi-material compound tool used as part of communicative behaviour. Although the function of excelsior in the tub-stacks is unclear, it is ostensibly a purposeful step of the behavioural sequence given that is present in 4 out of 5 videos. One possible function for the excelsior might be that it enhances the visual saliency of the display, as it can be seen to fly into the air when the tub-stack is struck. This may be analogous to the way branches are dragged or thrown by wild chimpanzees during their displays^64,65^. Another possibility is that the excelsior may mute or otherwise alter the acoustics of the tub in a way EH found pleasing. Alternatively, it may be that the excelsior cushions the impact of the tub on EH’s hands. As we can see from Videos 8–10, EH also strikes hard objects during their non-tub-stack pant-hoot displays (a steel sheet over a concrete wall), but it is possible that the use of excelsior nevertheless improves the comfort of the drumming and allows EH to strike the tub harder than they otherwise would. Multi-modal communication, where compound behaviours serve communicative functions by incorporating (for example) both vocal and gestural elements, has recently gained considerable interest as one means by which apes can maximise the expressive power of their limited repertoires^66^. Innovative use of tools or materials in this domain may be an additional means by which this communicative flexibility can be augmented.

Secondly, while chimpanzees are highly innovative, very few published examples related to the domain of communicative behaviour: across the 74 innovative behaviours collated in a recent cutting-edge review of chimpanzee innovation, only seven of these were related to communication^58^. Of these seven, just one involved a tool or object: a male chimpanzee incorporated petrol cans into impressively loud displays, to which the original authors attribute their rise to dominance in the group^43^ (Notably, EH was also the dominant male in their group). The tub-stack construction we describe is considerably more complex than this example, being comprised of a multi-step, multi-material process which *i*) typically begins before onset of the display it will be used to complement (likely requiring some degree of control over call production and/or forward planning, see below) and *ii*) requires an evaluation of the materials at hand, altering them if necessary (i.e. repairing the tub to make it viable as a drum). It is unclear whether the relatively few examples of communicative innovation in the literature are representative of a genuinely skewed distribution of the domains of behaviour in which chimpanzees possess the behavioural and cognitive flexibility to innovate, or instead reflect that innovations in the communicative domain have been overlooked by researchers (Bandini and Harrison’s review^58^ does not include, for example, the ‘raspberry blowing’ communicative innovation described by Hopkins et al.^67^). Leaf-clipping, where males strip leaves from branches with their teeth, represents one behaviour observed in the wild which incorporates an object with vocal behaviour when accompanying the preparatory phase of pant-hoot displays^68^. There is even some evidence that this behaviour may vary between communities in frequency and function, indicating a potential learned component (and hence potentially innovative in origins, rather than species-typical)^69^, but further work in this area is needed.

While chimpanzees can modulate the acoustic structure of some calls^22,23,70,71^, they are not vocal production learners^72,73^, and their gestural repertoire appears to be largely innate^74^, our observations indicate there may be a rich variety of other ways in which communicative behaviour can be augmented through behavioural flexibility and other innovations. Indeed, some studies have suggested that ‘buttress drumming’ in combination with pant-hoots varies between chimpanzee communities, indicating a possible cultural influence^39^. Recent work has also found that individuals also flexibly alter the rhythm of their drumming behaviour to provide cues to individual identity during travel behaviour, but not during displays^45^. We suspect that innovative behaviours in the communicative domain may have been overlooked, and encourage researchers to be forthcoming with any examples they have observed so that a more holistic understanding of behavioural flexibility in chimpanzees can be developed.

Two alternatives to EH innovating the behaviour entirely by themselves also exist: a) that EH learned it from observing a human, or b) that EH learned it from another chimpanzee. While the study site was not under constant surveillance, the results of our survey indicate it is highly unlikely EH ever observed a human interacting with a feeding tub in this way. The tub-stack behaviour has also never been observed in another chimpanzee at the study site, making conspecific social learning unlikely. However, given that EH’s behaviour was also unknown to care staff until our survey, we cannot rule out the possibility that it was more widespread and had simply gone unnoticed. Nevertheless, this would still mean the behaviour was innovated by some chimpanzee at some point, even if it was not by EH. Another possible explanation is that, while care staff at the facility had never observed the tub-stack behaviour before, and therefore could not have rewarded it directly, it is possible that individual elements of the behaviour (e.g. drumming, playing with excelsior) were directly or indirectly rewarded. EH could then have integrated these behaviours into a single sequence constituting the tub-stack. Alternatively, EH could have observed individual elements of the behaviour in other chimpanzees and integrated them into the tub-stack behaviour. However, both of these explanations nevertheless involve some degree of innovation in the combining of behaviours for novel purposes^58^, even if elements of the behaviour were reinforced or socially learned.

### Future planning

The ‘Bischof-Köhler’ hypothesis states that non-human animals are unable to dissociate from their current motivational state, and are therefore unable to anticipate future motivational states and plan for them^75–77^. More recent work has contradicted this hypothesis, demonstrating instances of forward planning in species including birds^78,79^, monkeys^80,81^ and apes^82–85^ (see Raby and Clayton^76^ for review). However, in each case, the behaviours identified were related only to foraging and tool-use behaviours rather than communication. The tub-stacking behaviour reported here is therefore particularly interesting because it may represent evidence of (albeit short-term) forward planning in the domain of communicative behaviour: EH intentionally organises their time to prepare an object (the tub-stack) which will be used to augment a future pant-hoot display. It is reasonable to interpret tub-stack construction as an intentional behaviour for several reasons. First, it involves the combination of multiple materials which could not plausibly repeatedly occur by chance. Second, it sometimes involves the transportation of those materials to another location (see Video 3). Third, in most cases, vocalisations do not begin until after the tub-stack is complete. Fourth, effective use of the tub-stack has prerequisites (the tub must be ‘in-tact’) which EH adapts their behaviour to meet if necessary. For example, in Video 1 EH does not need to repair the tub, so does not. In Video 2 it is necessary to repair the dent, so EH does. In Video 5 the tub is dented, and EH does not strike it as powerfully as when an in-tact tub is available. In this last case, it is unclear why EH does not repair the tub—we suggest that either the motivational state underlying their pant-hoot was too far along to suppress for long enough to create the tub-stack, or perhaps this video is unrelated to tub-stack behaviour and EH was simply drumming on a nearby object (which happened to be the tub).

The extensive capacity for mental time travel in humans confers many obvious benefits for problem-solving and coordination of behaviour, but has also been proposed as being a major driving force behind language evolution^86^ and indeed plays a crucial role in language processing^87,88^. EH’s behaviour suggests that animal communication may prove to be fertile ground for further examination of this topic, yielding potentially important insights into the evolutionary progression of the interaction of forward planning and communicative behaviour.

## Summary

In this report, we present an intriguing set of observations of a non-enculturated, captive male chimpanzee carrying out a multi-material, multi-step ‘preparatory phase’ to their pant-hoot displays. We offer several explanations for this behaviour, each of which would suggest a greater degree of voluntary control over the onset of vocal behaviour than has been typically described in chimpanzees^52–54^, and may even indicate a capacity for short-term forward planning. Significantly, as far as we know this is also the first recorded example of a chimpanzee engaging in communicative behaviour which makes use of a compound object or tool created from two different materials. Being based on a small number of observations from a single individual, we naturally advise caution in overinterpreting these data or generalising to other individuals, but we hope that this report stimulates further consideration of the ways in which vocal behaviour can interact with domains of cognition typically associated with foraging and tool-use in illuminating ways. Indeed, the carefully reported and interpreted anecdotal accounts of great ape behaviour have proven a rich source of insights into the cognition of these species^25,67,89^. Questions remain over the extent to which such insights can be generalised to wild populations, and this is likely to be a fertile area for more systematic observation and experimentation in the future.

## Ethical approval

The footage reported here was recorded during data collection for Watson et al.^48^, for which ethical approval was granted by the Institutional Animal Care and Use Committee of the UTMDACC, adhering to all the legal requirements of U.S. law and the American Society of Primatologists’ principles for the ethical treatment of nonhuman primates.

## Supplementary Information

Supplementary Information.

## Data Availability

The dataset described in the current study is available in the Open Science Framework repository at the following link: https://osf.io/w68fr/.
