# Supplementary material for: Innovative multi-material tool use in the pant-hoot display of a chimpanzee
Source: Sci Rep. 2022 Nov 29;12:20605. doi: 10.1038/s41598-022-24770-w (PMC9708694; doi:10.1038/s41598-022-24770-w)
Supplement: Supplementary file 1 — Supplementary Information. [file 41598_2022_24770_MOESM1_ESM.zip › Supplemental material - Raw answers.pdf]

- **Have you ever observed EH carry out the tub-stack behaviour (or variation on it) before this footage was recorded (Autumn, 2018)?**

R1: No

R2: I definitely recall seeing him display with the black tubs but cannot recall him using the excelsior. I also can't recall him fixing a dented tub.

R3: I do not recall seeing him carry out this specific behavior before the footage, however I have seen him use the black tubs for displays.

- **Have you ever observed EH carry out the tub-stack behaviour (or variation on it) in the years after this footage was recorded (Autumn, 2018)?**

R1: No

R2: Same answer as R1. I recall him using the black tubs during displays but can't recall him using excelsior. I also can't recall him fixing a dented tub.

R3: I do not recall seeing him carry out this behavior after the footage was recorded.

- **We are curious whether EH may have learned the tub-stack behaviour from watching a human. Can you think of any reason a keeper or trainer would have created something the 'tub-stack', or anything like it, in front of the chimpanzees?**

R1: No, there is no reason this would EVER be the case.

R2: I agree with R1.

R3: I agree with R1 and R2.

- **How many years have you personally known EH?**

R1: November 2006.

R2: I have known EH since Nov. 2005.

R3: I have known EH since June 1997.

- **Was the behaviour ever rewarded by people?**

R1: Not that I know of.

R2: I agree.

R3: I agree.

- **Did you ever observe the tub-stack behaviour in other individuals?**

R1: Not this exactly but [Another individual, not EH] will get a barrel before his display starts and makes sure it is in the position he wants it and where he wants it before he displays. I have seen many males do this over the years.

- **Have you noticed any similar behaviours at Bastrop? Specifically, either 'preparing' objects for a display (positioning them, combining them, etc)**

R1: If you are talking about preparations for displays ONLY then mostly just the barrel stuff I mentioned above.

R2: [Another individual, not EH] is one to do specific things to make the most noise during his displays. One thing that he does, if he has the option, is to pull down the guillotine door to bang on it. So he does manipulate the door with the sheer intention of using it in his display.

R3: I have also seen many chimps adjust items (mainly barrels) to just the right position to push around the enclosure during displays. Some of the chimps, like [two names], will go to specific places in their inside enclosure to bang consistently because it gives them the type of sound they are looking for. They don't adjust the cage of course, like EH adjusted the tub, but they are similarly performing their display with the sound level they desire. We also have these small plastic balls that are in the enclosures with the chimps as toys. We used to have a group that had two types of balls, one that was empty and one that had these metal pieces in it that kind of jingled when they shook it. We had one chimp that would always choose the ball with the metal pieces in it to wrack on the mesh during his displays since it made the most noise.

- **In a previous answer, you mentioned that some chimpanzees use water barrels in their displays. Have you ever observed EH doing this, or was it only in other individuals?**

R1: Yes, EH used the 55-gallon empty barrels in his displays when he was in a corral enclosure.

R2: I have also seen EH use these barrels during displays when he was housed in a dome.

R3: I have seen EH use barrels for displays as well.
